# Contrasting genetic diversity and population structure among three sympatric Madagascan shorebirds: parallels with rarity, endemism, and dispersal

**DOI:** 10.1002/ece3.1393

**Published:** 2015-01-23

**Authors:** Luke J Eberhart-Phillips, Joseph I Hoffman, Edward G Brede, Sama Zefania, Martina J Kamrad, Tamás Székely, Michael W Bruford

**Affiliations:** 1 Department of Animal Behaviour, Bielefeld UniversityMorgenbreede 45, 33615, Bielefeld, Germany; 2 Biodiversity and Ecological Processes Group, Cardiff School of Biosciences, Cardiff UniversityCardiff, CF10 3AX, U.K; 3 Department of Animal Biology, University of ToliaraPO Box 185, Toliara, Madagascar; 4 Biodiversity Lab, Department of Biology and Biochemistry, University of BathBath, BA2 7AY, U.K

**Keywords:** Abundance, dispersal, endemism, gene flow, genetic drift, shorebird

## Abstract

Understanding the relative contributions of intrinsic and extrinsic factors to population structure and genetic diversity is a central goal of conservation and evolutionary genetics. One way to achieve this is through comparative population genetic analysis of sympatric sister taxa, which allows evaluation of intrinsic factors such as population demography and life history while controlling for phylogenetic relatedness and geography. We used ten conserved microsatellites to explore the population structure and genetic diversity of three sympatric and closely related plover species in southwestern Madagascar: Kittlitz's plover (*Charadrius pecuarius*), white-fronted plover (*C. marginatus*), and Madagascar plover (*C. thoracicus*). Bayesian clustering revealed strong population structure in the rare and endemic Madagascar plover, intermediate population structure in the white-fronted plover, and no detectable population structure in the geographically widespread Kittlitz's plover. In contrast, allelic richness and heterozygosity were highest for the Kittlitz's plover, intermediate for the white-fronted plover and lowest for the Madagascar plover. No evidence was found in support of the “watershed mechanism” proposed to facilitate vicariant divergence of Madagascan lemurs and reptiles, which we attribute to the vagility of birds. However, we found a significant pattern of genetic isolation by distance among populations of the Madagascar plover, but not for the other two species. These findings suggest that interspecific variation in rarity, endemism, and dispersal propensity may influence genetic structure and diversity, even in highly vagile species.

## Introduction

It is well established that environmental barriers can restrict gene flow, facilitating genetic isolation by distance (Ehrlich and Raven 1969). Similarly, stochastic processes are known to interact with demographic characteristics, intensifying genetic drift, and affecting genetic diversity (Nei et al. 1975). Endemic organisms may be especially sensitive to the effects of isolation and genetic drift due to limited gene flow and typically small effective population sizes (Frankham 1997; Woolfit and Bromham 2005). Thus, population size, dispersal propensity, and endemism are presumed to be important drivers of population structure and genetic diversity (Frankham 1996, 1997; Freeland et al. 2011), yet few empirical studies have considered all three factors in concert. These factors go hand-in-hand in organismal biology and are important to understand for applications in conservation and evolutionary genetics.

Animal dispersal can be regulated by extrinsic factors such as geophysical processes (White et al. 2010) or niche gradients (Luppi et al. 2003), or by intrinsic factors such as breeding behavior (Greenwood 1980). Similarly, population size can be restricted by habitat and resource availability (Gregory and Gaston 2000) and niche tolerance (Brown 1984). Ecological specialists are typically range restricted and have low abundance (Brown 1984), making them relatively rare compared to generalists. Island endemism is also closely linked to rarity and dispersal propensity because it is predicted that island size and distance from the mainland will influence divergence times due to extinction and colonization processes, respectively (MacArthur and Wilson 1963; Johnson et al. 2000). However, these factors interact and vary across time, making it challenging to quantify and interpret their relative influence on gene flow and genetic drift. Comparative studies of multiple species offer a unique opportunity to explore how interspecific variation in rarity, endemism, and dispersal propensity shape comparative population genetic structure.

Most comparative population genetic studies of sympatric taxa have focused on marine organisms, where patterns are often attributed to differential dispersal opportunities via ocean currents (White et al. 2010). In terrestrial animals, similar comparative studies of sympatric taxa are rare and have focused mainly on ectotherms (e.g., Brede and Beebee 2003; Molbo et al. 2004; Manier and Arnold 2005). The vagility of birds inhabiting terrestrial environments is arguably analogous to the dispersal opportunities of organisms in the marine environment (Hillman et al. 2014). However, very few studies have been conducted on sympatric birds (Martinez et al. 1999; Smith et al. 2000; Petren et al. 2005) and ideally taxa should be selected for study that are both phylogenetically related and co-occur over the same geographic range.

Madagascar provides an excellent opportunity to investigate interspecific population genetic patterns because of its unusually high level of endemism – one in every 35 described vertebrate species on Earth is found only in Madagascar (Myers et al. 2000). This remarkable diversification of species has been attributed to the island's unique combination of an isolated geophysical history, steep gradients in local climate and habitat, and a tropical location (Vences et al. 2009). A convincing mechanism proposed to generate endemic biodiversity in Madagascar is the contraction and expansion of riverine habitats during Quaternary climate shifts, creating biotic refugia within isolated lowland watersheds (Wilmé et al. 2006). This “watershed mechanism” has been identified as an important process generating vicariant divergence in lemurs and reptiles throughout Madagascar (Wilmé et al. 2006; Pearson and Raxworthy 2009), but has not yet been tested on avian species. This is surprising considering that over half of Madagascar's birds are endemic (Goodman and Benstead 2005). However, much of the island is becoming increasingly threatened by habitat destruction through logging (Randriamalala and Liu 2010), mining (Cardiff and Andriamanalina 2007), and slash-and-burn farming (Styger et al. 2007), which have removed over 90% of the original primary vegetation (Myers et al. 2000). Consequently, Madagascar not only allows endemic and nonendemic species to be compared in sympatry, but is also important from a conservation perspective. Population genetic studies provide an important role in conservation biology by pinpointing genetically unique populations that require protection priority (e.g., Petit et al. 1998) and identifying species that have experienced population bottlenecks (e.g., Hoffman et al. 2011).

**Figure 1:**
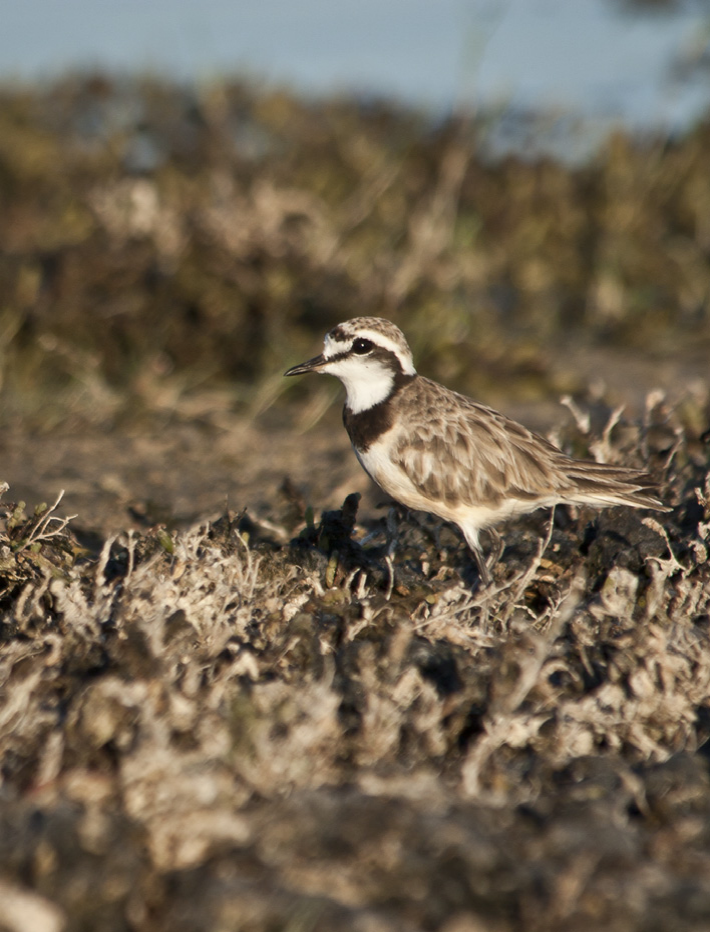
Adult Madagascar plover (*Charadrius thoracicus*) guards a nest at Andavadoaka, Madagascar (photograph by Luke Eberhart-Phillips).

Here, we took advantage of the highly tractable system provided by shorebirds of the *Charadrius* genus within Madagascar. Our aims were to quantify the population genetic structures and diversities of the Kittlitz's plover (*Charadrius pecuarius*), the white-fronted plover (*C. marginatus*), and the endemic Madagascar plover (*C. thoracicus*, Fig.1) and interpret these results in the light of interspecific differences in rarity, endemism, and dispersal propensity. All three plovers are sister species (dos Remedios 2013) that have overlapping distributions within Madagascar (Zefania and Székely 2013) which allows for comparisons of population structure and genetic diversity while controlling for phylogeny and geography (Bohonak 1999). Additionally, *Charadrius* plovers are easy to sample in the field (Székely et al. 2008) and microsatellite markers are well established (Küpper et al. 2007). We hypothesized that the Madagascar plover would have the lowest genetic diversity and highest population structure due to its endemic status, high site-fidelity, and small population size. By contrast, the Kittlitz's plover was predicted to have the greatest genetic diversity and a panmictic structure owing to its large population size, widespread distribution, and high dispersal propensity. We predicted the white-fronted plover to have moderate population structure and levels of genetic diversity because it is intermediate in many respects compared to the other two species. We also evaluated whether our genetic data conformed to expectations of the watershed mechanism that has been suggested to facilitate biodiversity of other Madagascan wildlife.

## Materials and Methods

### Study species

Between 10,000 and 20,000 Kittlitz's plovers inhabit Madagascar (Delaney et al. 2009), mainly occupying open coastal salt marshes and inland wet grasslands and riverbanks that are typically associated with grazing zebu cattle (*Bos primigenius indicus*; Appert 1971). On the other hand, about 5000–15,000 white-fronted plovers reside in Madagascar (Delaney et al. 2009), where inland populations are closely associated with riverine habitat and are less numerous than coastal populations which breed on open sections of sandy beach and salt marsh habitats (Zefania and Székely 2013). Phylogenetic evidence suggests that Madagascan populations of white-fronted plovers are genetically distinct from populations of mainland Africa, although Kittlitz's plover populations of Madagascar exhibit comparatively lower genetic differentiation from mainland populations than those of white-fronted plovers (dos Remedios 2013). Lastly, Madagascar plovers are endemic to the island and have the smallest population of the three species with a conservative estimate of 3500 individuals (Long et al. 2008). Madagascar plovers are restricted to sparsely vegetated shorelines of lakes and salt marshes within 10 km of the west coast of the island (Long et al. 2008). Because of their small population size, restricted range, and recent anthropogenic pressures on critical wetland habitats, Madagascar plovers are considered vulnerable (Long et al. 2008). In regions of Madagascar where the distributions of the three species overlap, Kittlitz's, white-fronted, and Madagascar plovers breed alongside each other in unison (Zefania and Székely 2013); however, the white-fronted and Madagascar plovers are socially monogamous (Zefania et al. 2010), whereas the Kittlitz's plover has low mate-fidelity (Parra et al. 2014) and a flexible mating system (Zefania et al. 2010). Between breeding seasons, marked Kittlitz's plovers in Madagascar have been resighted up to 113 km from where they were initially captured, whereas marked white-fronted and Madagascar plovers have not been resighted more than 15 km from natal sites (Zefania and Székely 2013).

### Field and molecular methods

**Figure 2:**
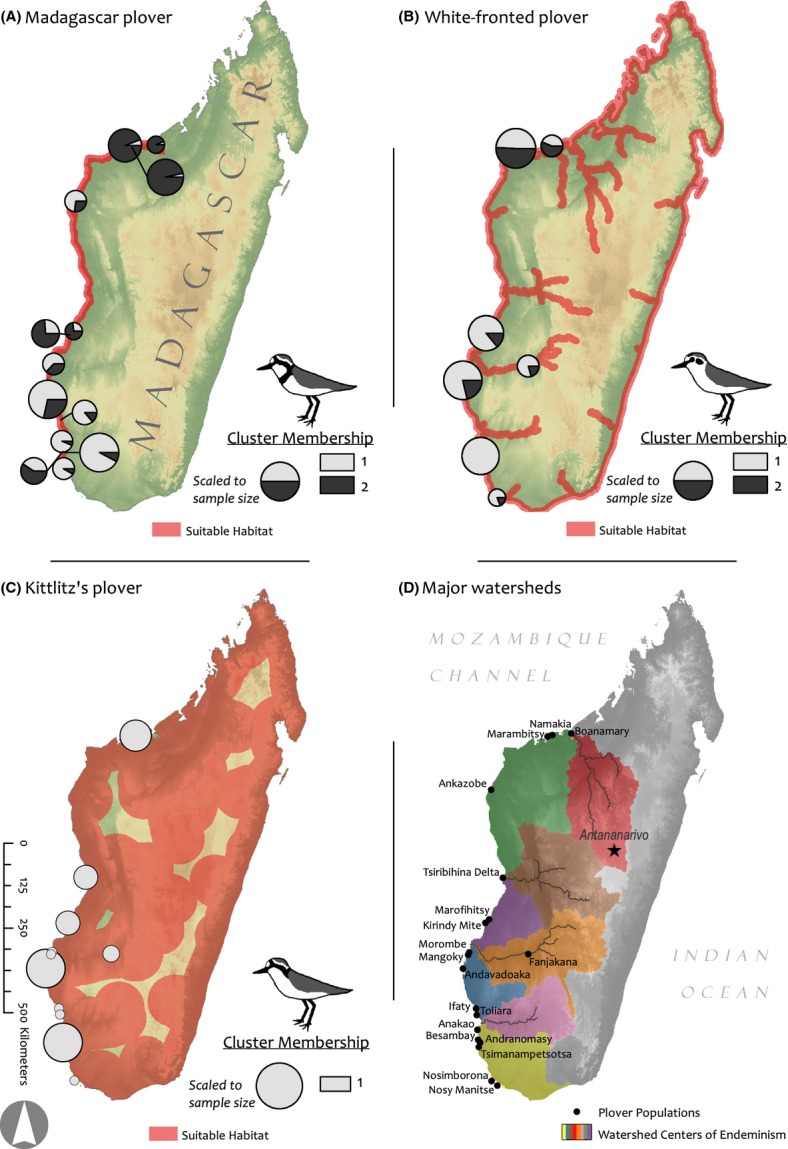
Results of STRUCTURE analyses for (A) Madagascar plover, (B) white-fronted plover, (C) Kittlitz's plover. Based on Δ*K* of the LOCPRIOR model, our genetic cluster analysis yielded a best estimate of two clusters (*K *=* *2) for the Madagascar and white-fronted plovers. Pie charts illustrate the proportion of sampled individuals from a given site that assign to each cluster. Panel D) illustrates sampled populations and watershed centers of endemism (colored polygons) within our study area as described by Wilmé et al. (2006). All panels are overlaid on topography (light green or gray) and the respective distribution of each species (red) as described by Zefania and Székely (2013).

We sampled a total of 114 Kittlitz's, 121 white-fronted, and 127 Madagascar plovers in 2010, from breeding sites along the western seaboard of Madagascar where the distributions of all three species overlap (Fig.2). Due to logistical limitations, our sampling effort was distributed ad hoc across known sites for each plover species. To control for confounding effects of geographic isolation and physical barriers to gene flow, we sampled as many species as possible from each site. Adults were captured on the nest using funnel traps, and approximately 25–50 *μ*L of blood was collected in capillary tubes after brachial venipuncture (Székely et al. 2008). Blood samples were stored in Queen's lysis buffer (Seutin et al. 1991).

Total genomic DNA was extracted using an ammonium acetate method (Bruford et al. 1998). From an initial set of 36 microsatellite loci that were shown previously to cross-amplify in a range of *Charadrius* species (Küpper et al. 2007), we evaluated the cross-species amplification of 18 loci in a subset of our samples from each species. Of these, 10 markers (*Calex-01, -06, -16, -19, -33, -35, -36, -43, -45, and -201*) amplified polymorphic and clearly interpretable PCR products in at least one of the species, seven of which amplified in all three species. These markers were subsequently screened across all 362 individuals in two multiplexes using the PCR conditions described by Küpper et al. (2007). PCR products were genotyped on an ABI 3730 capillary sequencer by Macrogen Inc. (South Korea).

### Genetic diversity

For each species, we used ARLEQUIN 3.5 (Excoffier and Lischer 2010) to calculate the allelic richness (*A*), and the expected (*H*_E_) and observed (*H*_O_) heterozygosity of each locus. Unbiased estimates of expected heterozygosity (u*H*_E_) were calculated to compensate for variable, and in some cases, small sample sizes (Nei 1978). Deviations from Hardy–Weinberg equilibrium (HWE) were evaluated using exact tests on the island-wide sample of each species. We established the significance of these tests by running the Markov chain algorithm with the dememorization number set to 10,000, the batch number as 1000, and the number of iterations as 10,000. We applied sequential Bonferroni adjustment of significance levels (Rice 1989) with *α *= 0.05 to correct for multiple testing across loci and species.

To account for variable sample sizes, we employed HP-RARE 1.0 (Kalinowski 2005) to calculate standardized allelic richness (*A*_R_) equalized to a sample size of four genes (i.e., our smallest sample size across populations) with rarefaction. Significant differences among species in each of the above measures of genetic diversity were identified using two-way analyses of variance (ANOVA) with locus and population specified as random factors. Species comparisons were further evaluated for significance with post hoc Tukey's tests. All analyses were tested at *α *= 0.05.

### Population genetic structure

We evaluated population genetic subdivision using Bayesian clustering of the microsatellite data in the program STRUCTURE 2.3.4 (Pritchard et al. 2000). This method estimates the likelihoods of varying numbers of genetically distinct clusters (*K*) in the sample by probabilistically assigning individuals to one or more cluster in a manner that minimizes each cluster's deviation from the Hardy–Weinberg and linkage equilibrium. Membership coefficients represent the assignment probability of each individual's genome to the *K* inferred clusters. We conducted these analyses using the LOCPRIOR model in STRUCTURE, for which we provided a priori information about the sampling locations of individuals across the study area (Hubisz et al. 2009). Assuming that individuals from the same sampling location have the same ancestry, the LOCPRIOR model prefers clustering scenarios that correlate with sample group identity. This way, the model allows for the detection of genetic subdivision even if population structure is weak, whereas it produces substantially similar outcomes as the uninformed model for strong structure signals (Hubisz et al. 2009). We ran five independent simulations using the admixture and correlated allele frequencies models for each *K* ranging from 1 to 20. For each run, we set the burn-in period to 10^5^ and used 10^6^ Markov chain Monte Carlo repetitions. We averaged the estimated likelihoods (Ln *P*[D]) of each *K* over the five independent runs and used both the maximum Ln *P*[D] and Δ*K* statistics to infer the most likely number of distinct clusters given our data. Δ*K* is an ad hoc statistic that uses the second-order rate of change of the likelihood function to reveal the relative amount of inference gained between successive *K* values (Evanno et al. 2005).

### Isolation by distance

For each species, we estimated pairwise genetic differentiation between locations using Wright's *F*-statistic (Wright 1949) calculated with ARLEQUIN. Significance of *F*_ST_ values was evaluated using 100 permutations of our data. To explore how geographic distance and the local environment explained genetic isolation by distance, we employed MRMPA (multiple regression matrix permutation using AIC; Kurvers et al. 2013). This was favored over a traditional partial Mantel test because it allowed us to control for more than one covariate. To acknowledge model parsimony, we first assessed a simple relationship between genetic differentiation and Euclidean distance before testing landscape-based models, which relied on more assumptions.

Our landscape models used the location pairwise *F*_ST_ matrix as the dependent variable and fitted a pairwise cost-weighted dispersal distance matrix, and Bray–Curtis dissimilarity matrices of local annual precipitation and isothermality (i.e., an index of constancy in local temperature [*mean diurnal temperature range*]*/*[*annual temperature range*]) as independent variables. We extracted these location specific climate data from interpolated bioclimatic surfaces provided by WorldClim (Hijmans et al. 2005). Cost-weighted dispersal distances between paired locations were calculated by creating cost rasters in ArcGIS (ESRI*,* Redlands, CA, USA) that were based on proximity to suitable habitat described in detailed species accounts (Zefania and Székely 2013). In brief, Madagascar plover dispersal corridors were restricted to habitats <10 km from the coast, white-fronted paths were restricted to habitats <10 km from major rivers and the coast, and Kittlitz's plover paths were restricted to habitats <75 km from major rivers and the coast (Fig.2). We used the Cost Distance Matrix tool in ArcGIS to compile a matrix of the pairwise cost distances among locations based on the species-specific cost rasters described above. In essence, this tool attempts to find the most cost-effective route between two locations given the length and habitat suitability of the route. To control for spurious relationships stemming from pairs of locations with low sample sizes, we weighted *F*_ST_ values by the total number of samples representing a given location pairwise comparison (Dumouchel and Duncan 1983).

We evaluated significance by calculating a model's AIC_C_ (Burnham and Anderson 2002) and comparing it to AIC_C_ statistics of 10,000 permuted models. This method randomly permutated the matrix rows of one independent matrix while holding other matrices constant. MRMPA permutes columns in the same order as row permutation to prevent impossible matrix configurations being produced (Kurvers et al. 2013). We repeated this procedure 10,000 times and tallied the proportion of permuted models that had smaller AIC_C_ values than the original model, which resulted in a *P*-value that we compared to *α *= 0.05. To account for model uncertainty and minimize the effect of uninformative parameters, we model-averaged beta coefficients using Akaike weights (Burnham and Anderson 2002; Arnold 2010). We inspected the residuals of significant models for normality with the Shapiro–Wilk test. All modeling and statistical procedures were implemented in R version “Pumpkin Helmet” (R Development Core Team 2014).

### Vicariant divergence among watersheds

We also evaluated the fit of our data to a simple model based on the watershed mechanism proposed to promote vicariant divergence after Quaternary climate shifts in Madagascar (Wilmé et al. 2006; Vences et al. 2009). This was assessed by grouping populations according to the watersheds delineated by Wilmé et al. 2006 (Fig.2D) and comparing the allele frequencies within and among watersheds using a hierarchical analysis of molecular variance (AMOVA). We tested for significance with 10,000 permutations in ARLEQUIN at *α *= 0.05 (Excoffier and Lischer 2010).

## Results

### Hardy–Weinberg equilibrium

**Figure 3:**
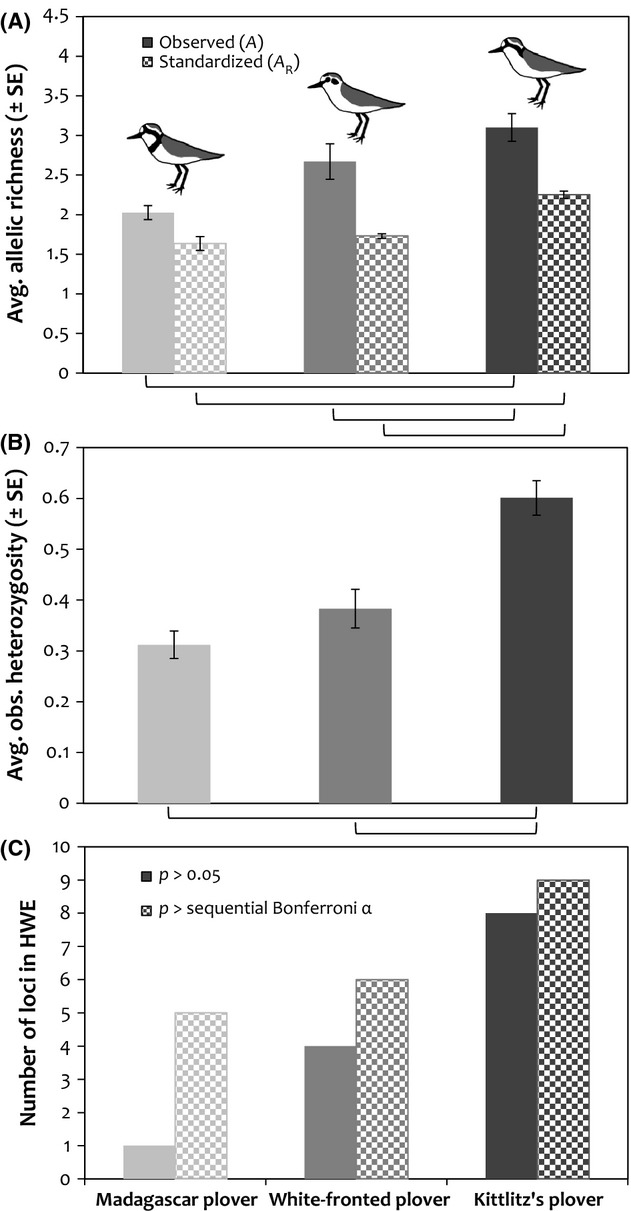
Interspecific variation in measures of genetic diversity, including (A) average observed allelic richness across populations (*A*, solid) and standardized allelic richness after rarefaction (*A*_R_, checkered), (B) average observed heterozygosity across loci and populations, and (C) number of loci in Hardy–Weinberg equilibrium at *α *= 0.05 (solid) and after sequential Bonferroni correction (checkered). Tukey's test comparisons significant at *α *= 0.05 are symbolized by brackets under each plot.

The number of loci deviating from HWE after sequential Bonferroni correction for multiple tests varied among the species at the island-wide sample. Four loci deviated from HWE in the Madagascar plover, two in the white-fronted plover and none in the Kittlitz's plover (Fig.3C) consistent with varying degrees of the Wahlund effect (Hartl and Clark 1998) at the island-wide scale.

### Genetic diversity

We found varying levels of genetic diversity in the three species, with the Kittlitz's plover carrying between 2 and 10 alleles per locus and *H*_O_ ranging from 0.104 to 0.961 (Table1), white-fronted plover loci carrying between 2 and 12 alleles with *H*_O_ ranging from 0.146 to 0.859 (Table1), and Madagascar plover loci carrying between 3 and 7 alleles with *H*_O_ ranging from 0.051 to 0.528 (Table1). There were significant differences among species in *A* (mixed effects ANOVA: *F*_2, 286_ = 29.15, *P *<* *0.0001; Fig.3A) and *A*_R_ (*F*_2, 286_ = 13.46, *P *<* *0.0001; Fig.3A), with white-fronted and Madagascar plovers having significantly lower allelic richness than the Kittlitz's plover for both *A* (Tukey's test: white-fronted/Kittlitz's *z*_s_ = −2.92, *P *=* *0.009; Madagascar/Kittlitz's *z*_s_* *= −5.14, *P *<* *0.001; Fig.3A) and *A*_R_ (white-fronted/Kittlitz's z_s_ = −5.65, *P *<* *0.0001; Madagascar/Kittlitz's *z*_s_* *= −7.15, *P *<* *0.0001; Fig.3A). Likewise, we detected significant differences in *H*_O_ among species (*F*_2, 286_ = 23.27, *P *<* *0.0001; Fig.3B), with white-fronted and Madagascar plovers having significantly lower heterozygosity than Kittlitz's plovers (white-fronted/Kittlitz's *z*_s_ = −4.58, *P *<* *0.0001; Madagascar/Kittlitz's *z*_s_* *= −6.59, *P *<* *0.0001; Fig.3B).

**Table 1:** Estimates of genetic variability in Kittlitz's (KiP), white-fronted (WfP), and Madagascar (MP) plovers sampled across western Madagascar and genotyped at 10 loci. Totals and averages (±SE) of each species are summarized in the bottom three rows

Watershed^1^	Population	UTM Coordinates^2^	Species	Individuals	*A*	*A*_R_	*H*_O_	u*H*_E_
Betsiboka	Boanamary	640484E, 8250571N	MP	2	1.67	1.67	0.33	0.37
Melaky	Namakia	585313E, 8242761N	MP	13	1.78	1.50	0.27	0.27
			WfP	3	2.10	1.81	0.40	0.39
			KiP	29	4.44	2.33	0.59	0.60
	Marambitsy	569852E, 8242662N	MP	21	2.22	1.49	0.26	0.25
			WfP	39	3.80	1.73	0.36	0.35
	Ankazobe	403262E, 8084241N	MP	3	1.44	1.41	0.30	0.24
Tsiribihina	Tsiribihina Delta	438410E, 7824207N	KiP	4	3.22	2.36	0.69	0.60
Menabe	Marofihitsy	397539E, 7700390N	MP	2	1.44	1.44	0.28	0.24
	Kirindy Mite	385316E, 7689513N	MP	6	1.89	1.58	0.32	0.30
			WfP	18	3.00	1.79	0.38	0.38
			KiP	5	3.11	2.23	0.58	0.56
Mangoky	Fanjakana	513668E, 7598845N	WfP	3	1.70	1.62	0.43	0.34
			KiP	3	2.22	2.22	0.44	0.65
Mikea	Mangoky	338220E, 7603585N	MP	3	1.89	1.65	0.33	0.31
			KiP	2	2.44	2.16	0.52	0.54
	Morombe	335142E, 7596698N	KiP	2	2.00	2.00	0.72	0.57
	Andavadoaka	320670E, 7555488N	MP	30	2.11	1.54	0.29	0.28
			WfP	32	3.60	1.74	0.38	0.35
			KiP	32	4.56	2.39	0.63	0.62
	Ifaty	358640E, 7437437N	MP	4	3.33	2.54	0.65	0.69
			KiP	2	2.00	2.00	0.44	0.54
	Toliara	361326E, 7418782N	KiP	2	2.44	2.44	0.61	0.59
Karimbola	Anakao	362863E, 7374809N	MP	3	1.44	1.33	0.15	0.18
	Besambay	365105E, 7344866N	MP	5	2.67	1.99	0.29	0.49
	Tsimanampetsotsa	370918E, 7341446N	MP	33	2.67	1.53	0.25	0.27
			WfP	26	2.70	1.62	0.33	0.31
			KiP	31	5.44	2.42	0.66	0.63
	Andranomasy	367452E, 7323842N	MP	3	1.78	1.58	0.33	0.30
	Nosimborona	404203E, 7223819N	KiP	2	2.22	2.22	0.72	0.57
	Nosy Manitse	421665E, 7209971N	WfP	2	1.80	1.80	0.40	0.37
5 Watersheds	13 Populations		MP	127	2.02 ± 0.09	1.63 ± 0.09	0.31 ± 0.03	0.28 ± 0.02
5 Watersheds	7 Populations		WfP	121	2.67 ± 0.23	1.73 ± 0.03	0.38 ± 0.04	0.36 ± 0.03
6 Watersheds	11 Populations		KiP	114	3.10 ± 0.18	2.25 ± 0.05	0.60 ± 0.03	0.59 ± 0.03

*A*: allelic richness; *A*_R_: standardized allelic richness; *H*_O_: observed heterozygosity; u*H*_E_: unbiased expected heterozygosity.

1 Watersheds are as defined by Wilmé et al. (2006).

2 UTM zone 38 south, TAN25.

### Population structure

Based on the LOCPRIOR analysis of our dataset in program STRUCTURE, the most likely number of genetic clusters identified by Δ*K* was two (LOCPRIOR: *K *=* *2, Figs1 and 3) for both the white-fronted and Madagascar plover. By contrast, we found no evidence of genetic clustering in the Kittlitz's plover (i.e., *K *=* *1, Figs1 and 3).

### Isolation by distance

Controlling for variable sample sizes, the Madagascar plover exhibited a significant isolation by Euclidean distance pattern (*p*_MRMPA_ = 0.050; Fig. 5A). The residuals of this model conformed to normality (Shapiro–Wilk: *W *=* *0.986, *P *=* *0.534) suggesting that the overall pattern is not driven by one or a small number of outliers. When we employed cost-weighted dispersal distance as a predictor of genetic differentiation, the pattern strengthened (*P *=* *0.010; Table2). No significant patterns in Euclidean or landscape models were obtained for white-fronted or Kittlitz's plovers (Fig. 5, Table2).

**Table 2:** Landscape-based isolation by distance analysis assessing the effect of cost-weighted dispersal distance (CWDD), dissimilarity in annual precipitation, and dissimilarity in isothermality on population pairwise *F*-statistics. Model-averaged results are shown below

Independent variable	Total AICc weight	Model-averaged beta coefficient estimate	Adjusted SE	*z*-value	*P*-value
Madagascar Plover					
CWDD	0.91	8.642e^−13^	3.339e^−13^	2.588	0.010
Annual Precipitation	0.34	4.945e^−2^	4.140e^−02^	1.194	0.232
Isothermality	0.18	−1.509e^−1^	3.201e^−01^	0.472	0.637
Kittlitz's					
CWDD	0.54	−7.806e^−10^	6.210e^−10^	1.257	0.209
Annual Precipitation	0.29	8.219e^−2^	5.843e^−02^	1.407	0.160
Isothermality	0.47	−4.772e^−1^	2.574e^−01^	1.854	0.064
White-fronted					
CWDD	0.36	−5.957e^−13^	5.626e^−13^	1.059	0.290
Annual Precipitation	0.38	6.289e^−2^	4.786e^−02^	1.314	0.189
Isothermality	0.19	2.600e^−1^	2.853e^−01^	0.911	0.362

### Vicariant divergence among watersheds

Across all three species, we found no significant differences in allele frequencies among the watersheds proposed by Wilmé et al. (2006) as centers of endemism (AMOVA: Madagascar plover, *F*_CT_ = 0.0269, *P *=* *0.0604; white-fronted plover, *F*_CT_ = 0.0316, *P *=* *0.203; Kittlitz's plover, *F*_CT_ < 0.001, *P *=* *0.494; Table3). Although allelic variation of the Madagascar plover among watersheds was only marginally insignificant at *α *= 0.05, variation among populations within watersheds (*F*_SC_ = 0.079, *P *=* *0.002; Table3) and also within populations explained a greater amount of variance in our data (*F*_ST_ = 0.104, *P *<* *0.001; Table3).

**Table 3:** Hierarchical analysis of molecular variance (AMOVA) results investigating the amount of allelic variation described within and among the watersheds defined by Wilmé et al. (2006)

Species/Variance Component	df	SS	Variance Component	Variation %	*P-*value
Madagascar plover					
Among watersheds (*F*_CT_)	4	17.177	0.0277	2.69	0.0604
Among populations within watersheds (*F*_SC_)	8	15.881	0.0793	7.69	0.00198
Within populations (*F*_ST_)	243	224.579	0.924	89.62	<0.0001
Total	255	257.637	1.0313		
White-fronted					
Among watersheds (*F*_CT_)	4	16.371	0.0582	3.16	0.202
Among populations within watersheds (*F*_SC_)	2	3.424	−0.00825	−0.45	0.441
Within populations (*F*_ST_)	239	427.533	1.789	97.29	0.0005
Total	245	447.329	1.839		
Kittlitz's plover					
Among watersheds (*F*_CT_)	5	17.025	−0.0233	−0.90	0.494
Among populations within watersheds (*F*_SC_)	5	14.822	0.0571	2.20	0.0750
Within populations (*F*_ST_)	217	555.333	2.559	98.70	0.00436
Total	227	587.180	2.593		

## Discussion

Our study quantified and compared the population genetic structures and diversities of three sympatric sister species of *Charadrius* plovers in Madagascar, a widely recognized hotspot for endemism and vicariant divergence. By sampling each species from the same geographic localities and genotyping them with a comparable panel of molecular markers, we could assume that our samples were equally exposed to geographic isolation and physical barriers to gene flow.

### Interspecific patterns of population structure and genetic diversity

**Figure 4:**
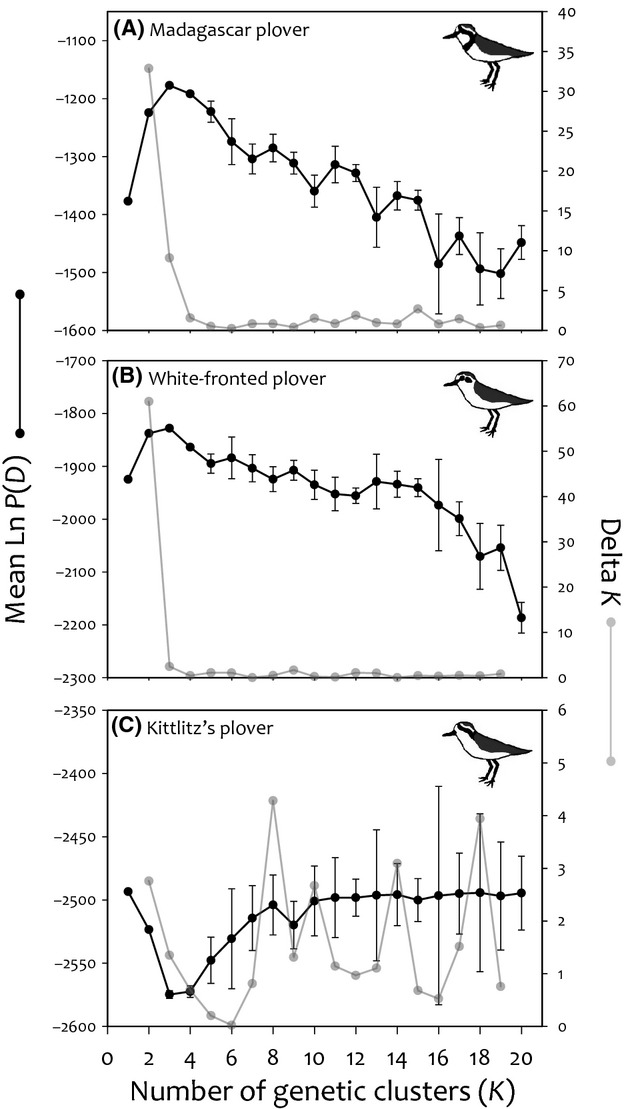
Mean of estimated Ln probability of the data [*P(D)*] (black) and Δ*K* (gray) at each potential number of clusters (*K*) using the LOCPRIOR model in STRUCTURE for (A) Madagascar plovers, (B) white-fronted plovers, and (C) Kittlitz's plovers.

We found contrasting patterns of population structure and genetic diversity among co-occurring Kittlitz's, white-fronted, and Madagascar plovers. Not only did we find stronger population structure in the Madagascar plover (Figs1A, 3A), but we also obtained a clear pattern of isolation by distance in this species (Fig.4A) that was lacking in the other two species (Fig.3B,C). We also observed a clear trend in genetic diversity across the three species (Fig.2A,B), with the Madagascar plover having the lowest allelic richness and heterozygosity, the white-fronted plover having moderate diversity, and the Kittlitz's plover having the highest allelic richness and heterozygosity. These contrasting patterns parallel interspecific trends in rarity, endemism, and dispersal propensity, which we propose may influence the population structure and genetic diversity of the three species.

### Rarity

Brown (1984) argued that generalist species are predicted to have large geographic distributions and to be locally abundant because they have the opportunity to populate a wider range of habitats than specialist species. Rarity – a combined measure of abundance and range size (Gaston 1994) – may therefore contribute to the influence of drift on genetic diversity. In our study, the Madagascar plover is a coastal specialist and is the rarest of the three species (Long et al. 2008), whereas the white-fronted plover is a semispecialist with moderate abundance, and the Kittlitz's plover is a generalist and relatively common (Delaney et al. 2009; Zefania and Székely 2013). The varying geographic distributions and abundances of these three species expose them to varying risks of local extinction and population bottlenecks because narrow distributions and small populations are more vulnerable to demographic and environmental stochasticity (Nei 1975, Johnson 1998). Therefore, our results follow the predicted relationship between genetic diversity and ecological niche tolerance, geographic extent, and abundance such that the Kittlitz's plover has the highest allelic richness and heterozygosity whereas the Madagascar plover has the lowest genetic diversity.

### Endemism

Theory and empirical evidence suggest that endemic island species tend to have lower genetic diversity than island species with mainland representatives (Jaenike 1973; Frankham 1997; Woolfit and Bromham 2005). This phenomenon is proposed to be a consequence of genetic drift and local adaptation (Jaenike 1973; Frankham 1997; Woolfit and Bromham 2005). Endemic island species typically have much earlier foundation times than nonendemic island populations (Frankham 1997). This may predispose small endemic island populations to the loss of genetic heterozygosity through drift (Frankham 1997; Woolfit and Bromham 2005). Likewise, natural selection for favorable alleles (or conversely, against unfavorable alleles) is predicted to increase loss of genetic diversity, assuming no heterozygote advantage (Frankham 1997). Recent phylogenetic evidence has revealed that the Madagascar plover colonized Madagascar approximately 6.6 Mya, followed by colonization of the white-fronted plover approximately 2 Mya, and the most recent colonization by the Kittlitz's plover <1 Mya (dos Remedios 2013). Thus, variation in endemism and colonization time among the three species could contribute toward interspecific variation in genetic diversity via genetic drift and potentially local adaptation.

### Dispersal

Gene flow can be regulated by extrinsic factors such as niche gradients (Luppi et al. 2003) or intrinsic factors such as breeding behavior (Greenwood 1980). Anecdotal observations of marked Kittlitz's plovers in our study area have been resighted up to 113 km from where they were initially captured in previous breeding seasons, whereas marked white-fronted and Madagascar plovers have not been resighted more than 15 km from natal sites (Zefania and Székely 2013). By implication, the Kittlitz's plover shows the greatest dispersal propensity at our study site. Although data on all three species are lacking for other locations, our resighting data for the white-fronted plover are supported by data from a population in mainland Africa (Lloyd 2008).

**Figure 5:**
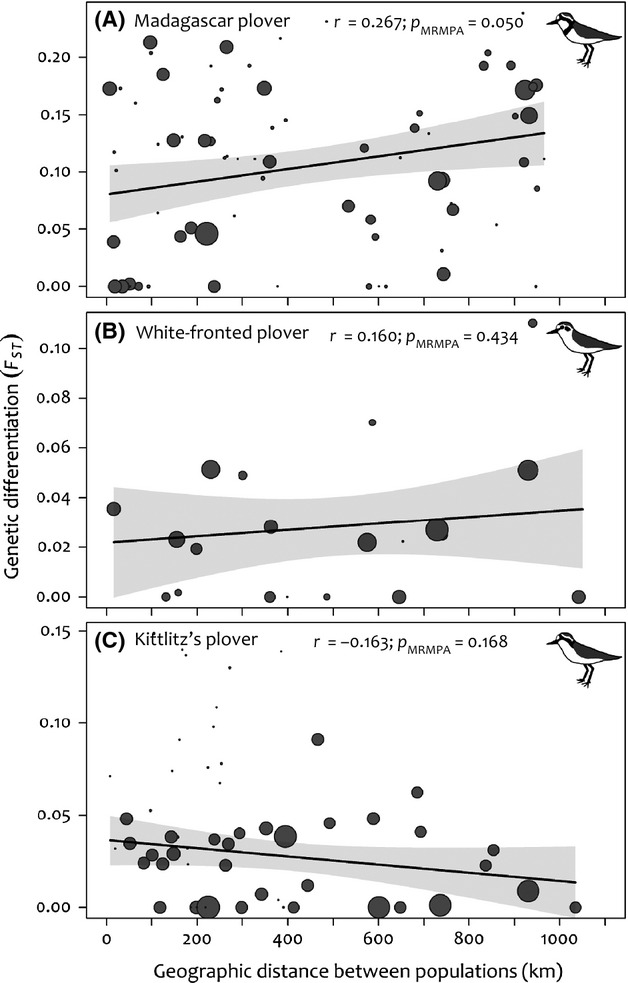
The relationship between geographic distance and genetic differentiation (*F*_ST_) among populations of (A) Madagascar plovers, (B) white-fronted plovers, and c) Kittlitz's plovers. Each circle symbolizes a pairwise comparison of two populations and is sized according to the number of samples representing a given pair of populations, which was used for weighting regressions. Solid gray polygons represent the 95% confidence interval of the linear regression and *P*-values based on MRMPA are reported.

Habitat generalists and specialists also differ in their opportunity to disperse (Zayed et al. 2005). This could potentially affect interspecific variation in gene flow depending on niche width. Our structure analysis yielded a clear north to south pattern in cluster membership of Madagascar plovers, with most individuals of the Marambitsy region in the north being assigned to a different cluster than those of the Tsimanampetsotsa region in the south (Fig.2A). This pattern was also apparent in the white-fronted plover, although not as strong (Fig.2B), whereas Kittlitz's plover populations appeared panmictic (Fig.2C). This pattern is consistent with the morphometric results of Zefania et al. (2010), who documented significant body mass differences between the northern and southern regions in both white-fronted and Madagascar plovers, but not in Kittlitz's plovers. Furthermore, the Madagascar plover showed a significant pattern of isolation by distance (Fig.5A), whereas we found no relationship between geographic distance and genetic differentiation in white-fronted or Kittlitz's plovers. Such a pattern could be attributable to differences in dispersal opportunity because habitat specialists such as the Madagascar plover tend to show reduced dispersal relative to habitat generalists (McCauley et al. 2014; Zayed et al. 2005; Kelley et al. 2000). This contention is also supported by our resighting data (Zefania and Székely 2013).

Alternatively, dispersal can be regulated by intrinsic breeding behavior. Mating system and parental care have been identified as important predictors of plover dispersal both within and between breeding seasons (Stenzel et al. 1994; Pearson and Colwell 2013) and natal site philopatry (Haig and Oring 1988; Colwell et al. 2007; Stenzel et al. 2007). It is therefore possible that breeding behavior could contribute to interspecific variation in population structure, either through variation in the tendency of individuals to divorce, disperse and find another mate, or via differences in natal philopatry. Among the three species in our study, the Kittlitz's plover is unique in that it has low mate-fidelity (Parra et al. 2014), uniparental care (Zefania and Székely 2013) and a flexible breeding system (Zefania et al. 2010). Conversely, white-fronted and Madagascar plovers have high mate-fidelity (Lloyd 2008; Parra et al. 2014), biparental care (Zefania and Székely 2013) and are socially monogamous (Zefania et al. 2010). It is expected that effective population size is higher for species characterized by monogamous mating systems than those that are otherwise-comparable but have less-monogamous breeding (Kaeuffer et al. 2007), which therefore might retain more diversity within populations and facilitate structuring in monogamous species. Likewise, a flexible mating system could cause more gene flow among populations than a less flexible system, which would reduce genetic differentiation among populations of polygamous species (Greenwood 1980).

### Watershed mechanism of vicariant divergence

The diversification of lemurs and reptiles among watersheds throughout Madagascar has been attributed to the contraction and expansion of riverine habitat during Quaternary climate shifts, which created biotic refugia within isolated lowland watersheds (Wilmé et al. 2006; Pearson and Raxworthy 2009; Vences et al. 2009). However, there are no studies addressing this phenomenon in Madagascan birds, despite the fact that most of the island's avifauna is endemic (Goodman and Benstead 2005). We did not find evidence of intraspecific vicariant divergence among major watersheds in Madagascar. In all three species, our AMOVA analysis found greater diversity within than among watersheds, with genetic differences between the watersheds being nonsignificant (Table1), suggesting that population structure is not consistent with the proposed watershed mechanism. We attribute this to the vagility of plovers (and birds in general), which likely facilitates greater gene flow among watersheds than other Madagascan organisms restricted to dispersal on land. Cowie and Holland (2008) reached a similar conclusion regarding endemic taxa of the Hawaiian Islands: Varying levels of vagility among taxa described differences in vicariant divergence within and between islands. Our study therefore suggests that the watershed mechanism may not be applicable to highly vagile species in Madagascar. This may have important implications for our broader understanding of Madagascan biodiversity.

### Caveats and conservation implications

Our study design, incorporating three sympatric and closely related species, allowed us to make broadscale inferences in respect to population structure and genetic diversity. However, the limited accessibility of sampling sites placed severe constraints on our sampling, particularly at remote locations. As a result, sample sizes were not always optimal, placing limitations on fine-scale inference. Nevertheless, in all three species, we were able to collect representative samples from at the very least the extremes (i.e., Namakia/Marambitsy and Tsimanampetsotsa) and center of the study area (i.e., Andavadoaka), revealing north to south gradients in the population structure of two of the three species. We also controlled for any potential biases resulting from variation in sample sizes by incorporating established statistical methods, such as unbiased estimations of heterozygosity (Nei 1978), rarefied allelic richness (Kalinowski 2005), and weighted linear regression (Dumouchel and Duncan 1983). The fact that our results are strong and consistent with expectations, despite these methods being highly conservative, suggests that the underlying patterns are robust.

Our findings also have important implications for plover conservation in Madagascar. The strong population structure of the Madagascar plover suggests that this species in particular may be vulnerable to inbreeding depression and the loss of genetic diversity owing to its low abundance and restricted distribution. Therefore, we advocate continued conservation of critical habitats of this vulnerable species to maintain sufficient genetic diversity needed to promote population viability.

## Conclusion

Using a comparative approach, we show that the Madagascar plover, an endangered endemic species with low abundance and a restricted range, is strongly structured and has low genetic diversity across its range. In contrast, the Kittlitz's plover, a widespread and abundant species with high dispersal propensity, is panmictic and has high genetic diversity over the same geographic area. The white-fronted plover, which is intermediate in many respects, exhibits moderate population structure and levels of genetic diversity. This pattern is consistent with what we know about these species' life histories, dispersal propensities, and endemic statuses. Thus, species traits may profoundly influence population structure and genetic diversity, with important implications for population, evolutionary and conservation biology.

## Data Accessibility

All relevant genotype and spatial data used to generate our results are publicly archived in Dryad (doi:10.5061/dryad.91b7m).
